# Monitoring Strategy for Eight Amphibian Species in French Guiana, South America

**DOI:** 10.1371/journal.pone.0067486

**Published:** 2013-06-28

**Authors:** Elodie A. Courtois, Jennifer Devillechabrolle, Maël Dewynter, Kévin Pineau, Philippe Gaucher, Jérôme Chave

**Affiliations:** 1 CNRS-Guyane USR 3456, Résidence Le Relais, Cayenne, French Guiana, France; 2 Station d'Ecologie Expérimentale du CNRS à Moulis, USR 2936, Saint Girons, France; 3 AGEP, Association de Gestion des Espaces Protégés, Maison des Associations, Bourg de Régina, Régina, French Guiana, France; 4 Biotope Guyane, Cayenne, French Guiana, France; 5 Laboratoire EDB UMR 5174, CNRS, Université Paul Sabatier, Toulouse, France; University of Kent, United Kingdom

## Abstract

Although dramatic amphibian declines have been documented worldwide, only few of such events have been quantitatively documented for the tropical forests of South America. This is due partly to the fact that tropical amphibians are patchily distributed and difficult to detect. We tested three methods often used to monitor population trends in amphibian species in a remote lowland tropical forest of French Guiana. These methods are capture-mark-recapture (CMR), estimation of the number of calling males with repeated counts data and distance sampling, and rates of occupancy inferred by presence/absence data. We monitored eight diurnal, terrestrial amphibian species including five Dendrobatidae and three Bufonidae. We found that CMR, the most precise way of estimating population size, can be used only with two species in high density patches where the recapture rate is high enough. Only for one of the species (*Dendrobates tinctorius*), a low coefficient of variation (CV = 0.19) can be achieved with 15 to 20 capture events. For dendrobatid species with day-calling males, audio surveys yield a better probability of detection with only 8 audio surveys needed; quantitative estimates can be achieved by computing the number of calling males inferred from audio counts or distance sampling analysis. We therefore suggest that an efficient monitoring protocol for Neotropical amphibian species should include a combination of sighting and audio techniques, and we discuss the need of implementing a large-scale monitoring in order to provide a baseline for comparison with future changes.

## Introduction

Amphibian populations are declining at an alarming rate across the globe [1], [2], with as much as one-third of extant species threatened with extinction. Although there is evidence that these declines are occurring worldwide, most of the studies come from Europe, North America and Australia [2]. Moreover, our lack of knowledge of pre-industrial population sizes creates the problem of shifting baselines in assessing the risk exposure for a species [3]. In order to evaluate the geographic extent of amphibian population declines and identify their causes, there is an urgent need for quantitative datasets in other parts of the world including South America [4]. The causes of amphibian population declines are likely multiple, including habitat loss, global climate changes, chemical pollution and the emergence of infectious diseases [5]. In the latter category, the fungus *Batrachochytrium dendrobatidis* (hereafter *Bd*) is one of the major threats to amphibians [6] and has been shown to drive declines even in pristine and remote areas [7].

Currently, 110 species of amphibians are recognized in French Guiana but true species richness is likely to be highest in this region [8]. The conservation status of amphibian species in French Guiana has been assessed on the basis of their estimated total population size and geographical distribution. Available data are not complete for most of the species and certainly not always informative of the threat of extinction [9]. Improving our knowledge of the conservation status of amphibian species in the Guianas is an ongoing research agenda that needs to be supplemented with rigorous long-term monitoring programs and observational data on population trends. Nonetheless, estimating absolute density and abundance of wild animals is particularly challenging when the probability of detection of the species is low, as it is the case for most tropical amphibian species [10].

Several methods have been proposed to monitor amphibian population trends [11]–[12]. Quantitative estimation of population size may be achieved via capture-mark-recapture (CMR) studies. CMR models rank among the most accurate techniques for population monitoring, but acquiring the underlying data needed for the use of these models is often both time consuming and expensive [13]. Indeed, CMR models require the estimation of individuals detection probabilities which can be achieve only with a sufficient recapture rate [14]. If the target species is inherently rare, and if the individuals are elusive, CMR is therefore difficult to use to infer total population size without a high capture effort [13].

Another population size index related to population abundance is obtained through individual counts, either through visual encounters [15]–[16] or audio detection [17]. In the latter case, repeating counts in multiple occasions and multiple sites allows taking into account imperfect detection [18] and estimating the number of calling males. Counts data were previously shown to provide a good estimate of population size for territorial frogs [19] and has been used in tropical forest to compare abundance of *Eleutherodactylus* frogs in a gradient of forest alteration [17]. Distance sampling may also be used to estimate calling males density using transects after accounting for detectability [20]–[21]. In this case, the observer travels along a line, recording all detected individuals and measuring the perpendicular distance of each to the center line. An important point about these methods is that only a fraction of the population is detectable using acoustics (only calling males) and care is needed to link these results with overall population estimates.

When quantitative estimation of population size or density cannot be easily achieved, another possibility to track trends in population size is to rely on indices of patterns of species presence across sites. In this case, presence/absence data are recorded and allow to follow the proportion of area (or sites) occupied by a given species [22]. Such models account for the fact that the detection probability of target species is generally less than one [22]. Thus, detection/non detection data are used to estimate the probability of occupancy corrected by the probability of detection of the species in a given habitat [22]. Briefly, the field sampling involves visiting sites multiple times and recording whether the target species is detected or not. The detection probability is then used to estimate the proportion of sites that are occupied by the species.

We conducted a pilot study in order to compare the feasibility of three methods used to monitor trends in amphibian populations over time for eight species in a remote tropical forest in French Guiana. Specifically, we contrast results from (1) quantitative population size estimation via CMR, (2) occupancy rate inferred from detection/non detection data and (3) estimation of the number of calling males by repeated counts and distance sampling. Based on this empirical evidence, we then assess the most suitable method for a comprehensive population monitoring of amphibians across Amazonia, and discuss the possible implementation of such a cross-continental monitoring program.

## Methods

### Studied Species

We monitored eight terrestrial, diurnal amphibian species, including five Dendrobatidae species (*Dendrobates tinctorius*; *Ameerega hahneli; Allobates femoralis*; *Allobates granti* and *Anomaloglossus baeobatrachus*) and three Bufonidae species (*Rhinella margaritifera, R. lescurei* and *Atelopus flavescens*). *A. flavescens* is listed as Vulnerable on the International Union for Conservation of Nature list and *A. baeobatrachus* and *R. lescurei* as Data Deficient (IUCN, 2011). The other five species are currently listed as Least Concern. In four out of the eight species studied (*A. hahneli, A. femoralis, A. granti* and *A. baeobatrachus*) males call from individual places during the day. Size and coloration vary greatly across species (Table 1).

**Table 1:** Description of the species monitored in this study and possible use of the methods used in this study.

Species	Mean Snout-Vent-Length (SVL)	Pattern used forindividual recognition	CMR	Occupancy rate	Number of calling males	Distance sampling	Most promising monitoring strategy
***Dendrobates tinctorius***	43	dorsal, ventral and lateral pattern	**Yes**	Visual	No	No	CMR
***Ameerega hahneli***	23	ventral pattern	No	Audio	**Yes**	**Yes**	Occupancy rate and Number of calling males
***Allobates femoralis***	20	ventral pattern	No	Audio	**Yes**	**Yes**	Occupancy rate and Number of calling males
***Allobates granti***	16	lateral band	No	Audio	**Yes**	No	Occupancy rate and Number of calling males
***Anomaloglossus baeobatrachus***	15	lateral band	No	Audio	**Yes**	No	Occupancy rate and Number of calling males
***Rhinella margaritifera***	57	dorsal and ventral pattern	**Yes**	Visual	No	No	Occupancy rate
***Rhinella lescurei***	36	dorsal and ventral pattern	No	Visual	No	No	Undertermined
***Atelopus flavescens***	36	dorsal pattern	No	Visual	No	No	Undertermined

### Study Site

**Figure 1:**
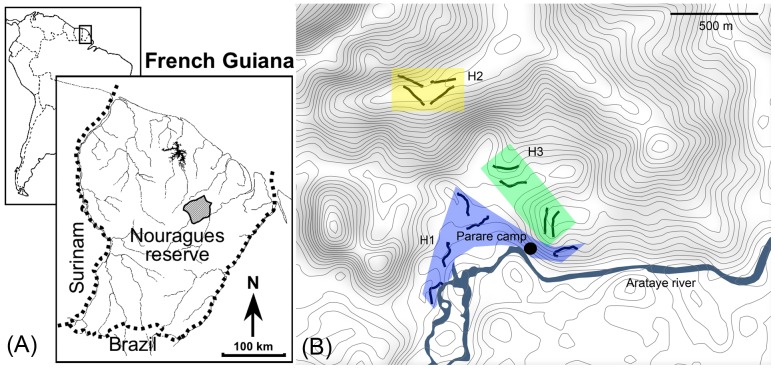
Location of the study site. (A) Location of the Nouragues reserve in French Guiana, and (B) Location of the Pararé site within the Nouragues Biological station (located inside the Nouragues reserve) with the 13 transects of 150 m in Habitat 1 (H1 in blue, along the Arataye river), Habitat 2 (H2 in yellow, in the plateau) and Habitat 3 (H3 in green, in the slope between the river and the plateau).

This study was conducted in French Guiana, at the Nouragues Biological Research Station, at the Pararé site (3°59′N, 52°35′W). This research station is located along the Arataye river within the Nouragues Natural Reserve (Figure 1-A). All necessary permits were obtained through a convention with the curator of the reserve (ONF Guyane). Mean annual temperature is 26°C and annual rainfall is 2990 mm, with a two-month dry season in September and October (though a drying trend is usually detectable early in August). The forest is an undisturbed lowland moist tropical forest, with canopy height ranging between 35 and 45 m above ground. Our study zone represents an area of 1.2 km^2^ (120 hectares, Figure 1-B). Field work was conducted from March 17^th^ 2011 to August 3^rd^ 2011 (rainy season and beginning of the dry season). Data from this study are available by contacting the corresponding author.

### Sampling Protocol

We defined three habitat types (Figure 1-B). The first habitat (H1) is the riparian vegetation along the Arataye river, at a mean elevation of 20 m asl. The second habitat type (H2) is the plateau forest at a mean elevation of 160 m asl. Finally, the third habitat (H3) is located along slopes at an intermediate elevation, between 57 and 72 m asl. We established and monitored a total of thirteen 150-m long linear transects, four in habitats H2 and H3, and five in habitat H1 (Figure 1-B). The distance between transects varied from 60 to 150 m. Visual and audio sessions were conducted from March to July 2011 with an average of 5 walks per month for visual counts and 4 walks per month for audio counts, for a total of 25 walks for each transect in H1, 27 walks in H2 and 28 walks in H3 (Table 2). Thus, over 50 km of transect were walked to generate this dataset.

**Table 2:** Number of audio and visual walks per transect for each month in each habitat and the total number of walks for each transect for visual and audio detection.

		March	April	May	June	July	Total
**Visual**	**H1**	5	2	3	7	8	**25**
	**H2**	5	4	5	5	8	**27**
	**H3**	5	3	5	7	8	**28**
**Audio**	**H1**	2	0	1	7	8	**18**
	**H2**	2	0	7	5	8	**22**
	**H3**	2	2	2	7	8	**21**

Each survey of the 150-m long transect lasted 30 minutes and each caught individual of the eight focal species was measured, weighed and photographed with a digital camera to record their dorsal, ventral and lateral color patterns (see Table 1 for details on each species). Within each species, individuals were recognized with their natural unique color patterns [23]–[24].

At the beginning of the study (March, April and May), audio sessions were decoupled from the counting sessions and in June and July, audio surveys were performed at the same time as visual transects. A total of 18 walks were therefore conducted for each transect in H1, 22 walks for H2 and 21 walks for H3 (Table 2). Each of these sessions lasted also 30 minutes and all calling males from *A. hahneli, A. femoralis, A. granti* and *A. baeobatrachus* were located, if heard, along the transect.

### Statistical Methods

For CMR, encounter histories were constructed for each individual on each transect based on whether they were detected (1) or not (0) in each sampling event. To evaluate the feasibility of Capture-Mark-Recapture (CMR) analysis, we first computed the recapture rate for each species in each habitat. For species with a recapture rate greater than 10%, we tested whether the populations were closed using the software CloseTest [14]. Selection of the best model for population size estimation was done using CAPTURE software included with program MARK [25]. All the models implemented in the software were tested and their likelihood was compared to the null model M(0) where capture probability is independent from temporal, individual and behavioral factors. In order to test how many encounter events are necessary for a confident estimation of population size, we used the package RMark [26] implemented in the R software (http://cran.r-project.org/), which allows estimating the population size (N) and its standard error for a growing number of encounter events (from 10 to the total number of encounter events). For this, we first estimated the population size (N) using only the first ten encounter occasions and then added sequentially the following encounter occasions, estimating at each step the population size and its standard error.

For occupancy rates, [22] described a likelihood-based method for estimating the proportion of area (or sites) occupied when species detection probabilities are less than one. We defined one area unit (or site) as one transect. Naïve occupancy (i.e. the percentage of sites where the species has been detected at least once) provides a lower bound as it assumes that the species has not been missed at any of the occupied sites. For each species, detection/non detection history was then used to estimate a constant detection probability (p) and a constant occupancy rate (ψ) using the package unmarked [27] in the R software.

Counts data using audio transects were analyzed with the Royle’s N-mixture models [18] implemented in the package unmarked [27] in the R software. Briefly, N-mixture models use spatial replication of repeated counts data to estimate the site-specific number of individuals (N) in the site. Spatial replications at more than one site give information about the distribution of N and leads to reasonable estimates of abundance even from scarce data [18].The males of dendrobatid species are territorial and calling from isolated places therefore we are confident that each male was counted only once on each walk. In these models, the abundance of each species at each site is assumed to remain constant in the time required to complete the survey. We don’t have such information for all the species but at least for one of them (*A. femoralis*), males display a high degree of site fidelity during the breeding season [28]. Moreover, site-specific abundances are supposed to follow a Poisson or negative binomial distribution. A negative binomial distribution fit the data well for three species among four (Goodness Of Fit test P = 0.06 for *A. femoralis*, P = 0.21 for *A. baeobatrachus*, P = 0.1 for *A. hahneli*), whereas a Poisson distribution did not fit the data (GOF test P<0.001 for the three species). For one of the species (*A. granti*), neither negative binomial distribution (GOF test p = 0.005) nor poisson distribution (P<0.001) fit the data well. We therefore used negative binomial distribution in the N-mixture models but results for *A. granti* need to be taken with precaution.

Concerning the distance sampling analysis we used the data for March where each transect have be walked twice totaling 61 observations for *Allobates femoralis* and 40 for *A. hahneli*. For *A. granti* and *A. baeobatrachus* the number of records during this month (N = 12 and 11 respectively) are not sufficient to use distance sampling analysis. Field data were subject to rounding to build 5-m classes of intervals. For *A. femoralis*, we truncated the data beyond 40 m (N = 2) and for *A. hahneli* beyond 20 m (N = 1). We compared uniform, half-normal, and hazard-rate key functions and used Akaike’s Information Criterion (AIC) to select the best model [20].

### Power Analysis

Using occupancy rates and detection probabilities estimated in this pilot study, we propose survey design recommendations [29]. First, we determined for each species the number of survey visits (K) required to determine species presence at an occupied site with a given probability. The probability of detecting the species when it is present in at least one of the visits is 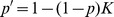, where p is the detection probability on a given walk (or visit). The number of visits (K) needed to achieve a given probability of detecting the species at least once (p’ = 0.8, 0.9, 0.95) is expressed as 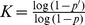.

Using again occupancy rates and detection probabilities estimated in this study, we asked which design for future surveys would allow detecting a decline in occupancy rate for each of the species [28]. We supposed a standard monitoring design with S sites sampled K repeated times (value of K determined before to achieve a p’ = 0.95). Based on this number of walks, we determined the number of sampling sites needed in order to detect a decline in occupancy rate between two surveys for a given power given an actual proportional decline (effect size) R. Power is defined as the probability to detect an actual decline (1-β) where β is the probability to conclude that there are no declines when a decline actually exists. The number of site required [28] is then defined as 
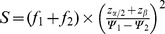
 where ψ1 is the initial occupancy rate and ψ2 is the occupancy rate after a proportional change R defined as ψ_2_ = ψ_1_ (1– R ). Terms f_1_ and f2 are defined respectively as f_1_ = ψ_1_ (1–ψ_1_+F) and f_2_ = ψ_2_ (1–ψ_2_+F) with F tending to zero as the probability of missing the species at occupied sites 1-p’ tends to zero. Finally, z_i_ is the value corresponding to α and β errors. We assessed three effect size (R = 5%, 15% and 30%) using a significance level α = 0.05. We tested the study design considering that the survey would be the same for multiple species. We assumed that detection probability and the number of replicate surveys were the same in the two sampling occasions. We used 5000 simulations and in each simulation two detection histories (with ψ1 and ψ2) were generated and power was computed as the proportion of simulations in which a significant decline was detected. For these simulations, we used the R code implemented in [30].

## Results

### Total Counts

A total of 350 visual observations have been made during the study including 180 observations (51%) of *D. tinctorius* and 81 observations (23%) of *R. margaritifera*. Using audio detection, 395 records were made, with 220 records (56%) of *A. femoralis* and 104 (26%) of *A. hahneli*.

### Population Size Estimates Using CMR

**Figure 2:**
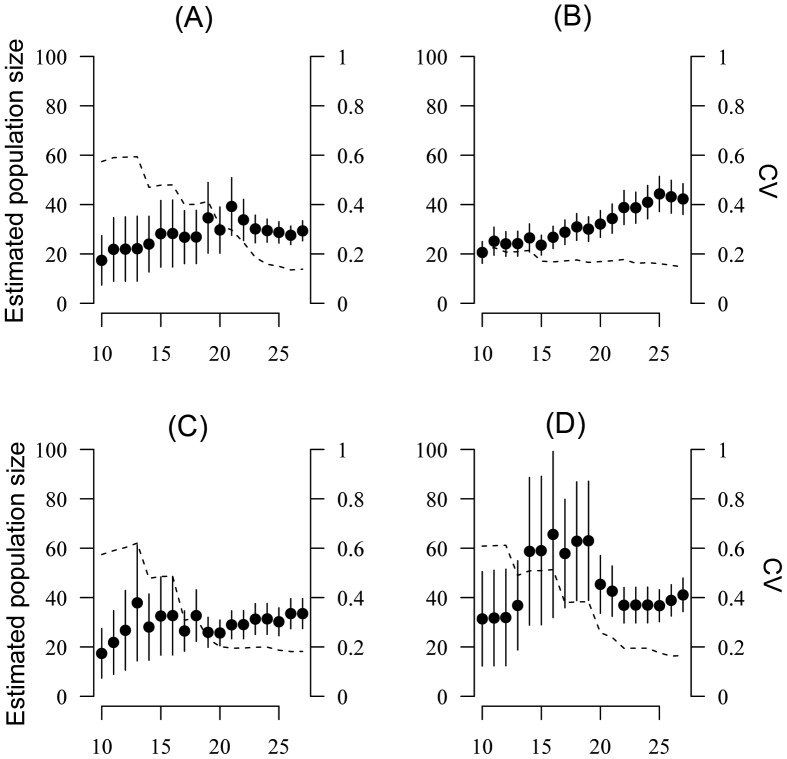
Estimation of *Dendrobates tinctorius* population size with increasing number of capture occasions. Evolution of population size (N, right axis) and standard deviation around the mean value estimated for *D. tinctorius* in habitat H2 by increasing the number of capture occasions for each of the four transect in habitat H2 (A) TH2A, (B) TH2B, (C) TH2C and (D) TH2D. Dotted lines represent variation of the coefficient of variation CV (left axis).

Recapture rates were greater than 10% only for *D. tinctorius* in the habitat H2 (recapture rate = 43%; 4 transects; Table S1) and for *R. margaritifera* in habitats H1 and H3 (10% and 13% respectively; 6 transects; Table S1). For *D. tinctorius*, 166 observations on 180 (92%) were made within a strip of 1.2 meter on each side of the transect. Within this area, the number of capture events probability of capture can be considered as uniform (Figure S1) and we therefore truncated the data to take into account only recaptures made within 1.2 meter on each side of the transect. When considering each transect separately, all populations can be considered as closed (Stanley and Burnham closure test, F = 20.5 and p = 0.3 for TH2A; F = 15.5 and p = 0.75 for TH2B; F = 15.2 and p = 0.65 for TH2C; F = 25.9 and p = 0.08 for TH2D). For 2 transects, the best model was the null model M(0) while for the 2 others transects, the best model was M(h), the Jackknife model including heterogeneity in capture probabilities among individuals (Table 3). Population size ranged from 34 to 47 individuals per transect (mean = 39.5 individuals per transect, SD = 7.8, Table 3). By considering that the sampling area is 1.2 meter on each side of the transect, *D. tinctorius* density can be estimated at 1097 individuals/Ha (39.5 individuals for 150*1.2*2 = 360 m^2^, Table 3). For *D. tinctorius*, the coefficient of variation (CV) with the maximum number of sampling event (27 sampling events) ranged from 0.18 to 0.21 (mean = 0.19). A stable estimation of population size (N) with a CV lower than 0.20 can be achieved for *D. tinctorius* with 15 to 20 sampling events (Figure 2).

**Table 3:** Density estimates using CMR models indicating the model used to make the estimation, the population size (N) estimated and its standard deviation, the coefficient of variation (CV), the confident interval at 95% for N, the capture probability for each transect and finally the density retrieved from the population size estimated via CMR.

Species	*D. tinctorius*				*R. margaritifera*					
Habitat	H2				H1			H3		
Transect	TH2A	TH2B	TH2C	TH2D	TH1A	TH1D	TH1E	TH3A	TH3C	TH3D
**Best model**	M(h)	M(o)	M(o)	M(h)	M(h)	M(o)	M(o)	M(h)	M(o)	M(o)
**N**	34	47	34	43	40	13	13	4	13	50
**SD (N)**	6.25	9.93	5.98	8.82	12.55	9.70	9.70	2.57	9.77	43.78
**CV**	0.18	0.21	0.18	0.21	0.31	0.75	0.75	0.64	0.75	0.88
**IC 95%**	27	35	27	34	25	7	7	4	7	17
**IC 95%**	54	77	52	71	77	47	47	21	57	238
**Capture probability**	0.05	0.03	0.04	0.04	0.02	0.19	0.19	0.05	0.02	0.01
**Density (N/Ha)**	944	1305	944	1194	1333	433	433	133	433	1666
**Mean density (N/Ha)**	1097				733			744		

For *R. margaritifera*, all the observations were made within a strip of one meter on each side of the transect and within this strip, the probability of capture cannot be considered as uniform (Figure S1). The estimated population size is therefore likely to be biased but if we truncate the data, recaptures rates are too low to run CMR methods. The null model M(0) was the best model for 4 of the 6 transects and the Jackknife model M(h) including heterogeneity in capture probabilities among individuals was used for the 2 remaining transects (Table 3). The average density of *R. margaritifera* was 740 individuals/Ha (733 individuals/Ha in habitat H1 and 744 individuals/Ha in habitat H2). The coefficient of variation with the maximum number of transect remained high (mean value of CV = 0.68).

### Determination of Occupancy Rate Using Detection/non Detection Data

**Figure 3:**
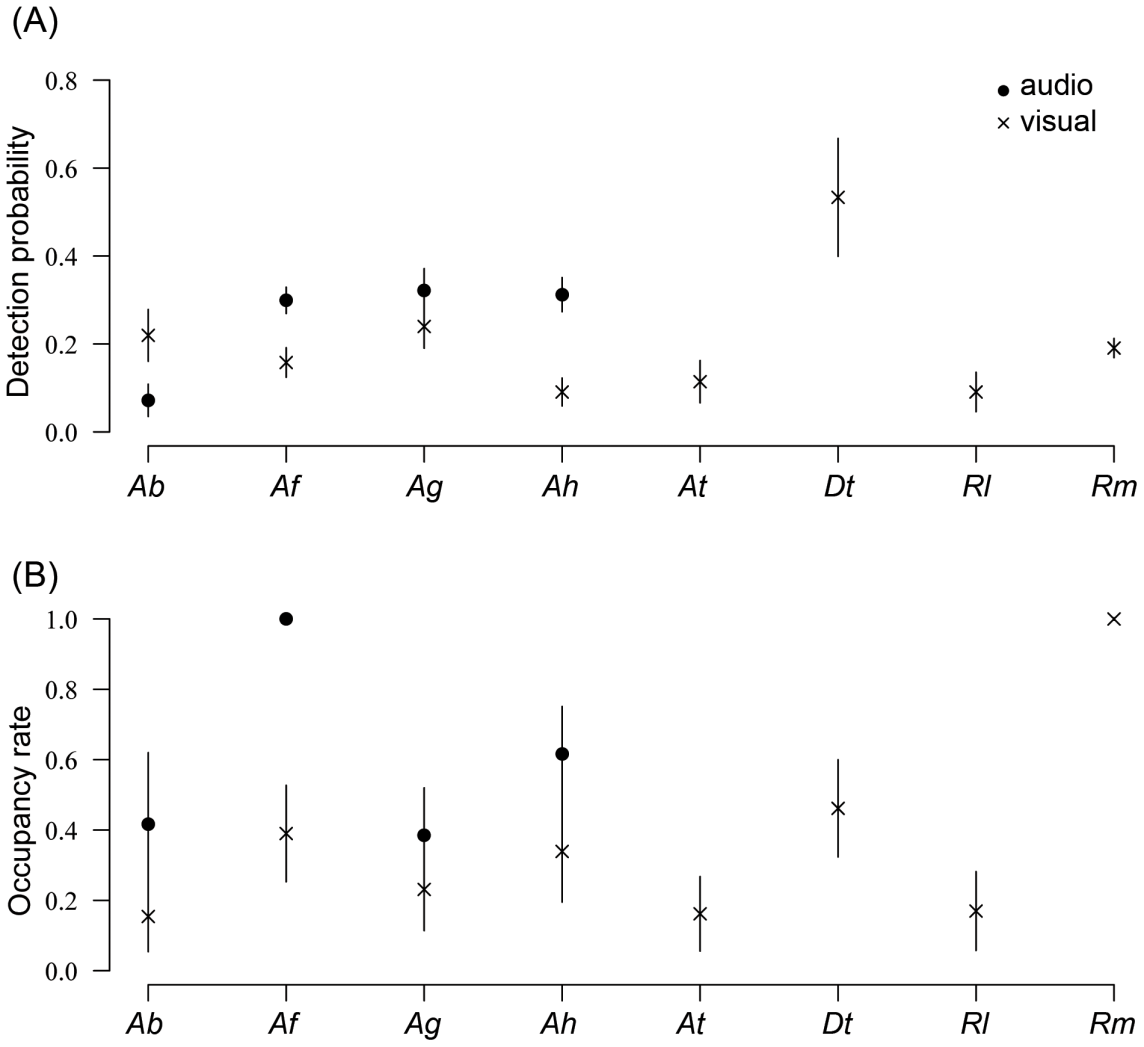
Probability of detection and occupancy rate. Estimations of (A) probabilities of detection and (B) occupancy rates for the eight species studied and standard deviation around the mean value for visual (points) and audio (cross) detection for Ab – A. baeobatrachus, Af – *A. femoralis*, Ag – *A. granti,* Ah – *A. hahneli*, At – *A. flavescens*, Dt – *D. tinctorius*, Rl – *R. lescurei*, and Rm – *R. margaritifera*.

The naïve occupancy (proportion of sites where the species has been detected at least once) was in all cases very close to the estimated occupancy (Table 4) suggesting that the species had been detected in most occupied sites, i.e. that p’ (probability to detect the species at least once) was close to one. Detection probabilities were always lowers than 0.60 irrespective of the species (Figure 3). Apart from *A. baeobatrachus,* the probability of detection was higher with audio than with visual detection for species with day-calling males (Figure 3). The species with the best probability of detection was *D. tinctorius* (probability of detection = 0.53+/−0.13; Figure 3). Occupancy rates were highest for *A. femoralis* (1+/−0.002 using audio detection) and *R. margaritifera* (1+/−0.003 using visual detection). Other Dendrobatidae species displayed occupancy rates close to 0.5 (0.39+/−0.14 for *A. granti*, 0.41+/−0.20 for *A*. *baeobatrachus* and 0.61+/−0.14 for *A. hahnelli* estimated with audio detection; and 0.46+/−0.14 for *D. tinctorius* estimated with visual detection; Table 4).

**Table 4:** Results from occupancy modeling (ψ, occupancy rate and p probability of detection) for the eight species with the type of detection used (A for Audio and V for Visual) and results from the power analysis with the estimated number of visits (K) needed to achieve a p’ (probability that the species is detected at least once during the survey) equal to 0.8, 0.9 or 0.95 and the number of sites (S) needed with 8 visits (K = 8) to detect a decline of R equal to 0.5, 0.15 or 0.30.

Species (Detection)	Naïve occupancy	ψ (SD)	p (SD)	K (p' = 0.8)	K (p' = 0.9)	K (p' = 0.95)	S (R = 0.05)	S (R = 0.15)	S (R = 0.30)
*A. femoralis* (A)	1.00	**1** (0.002)	**0.30** (0.03)	4	6	8	835	127	40
*A. granti* (A)	0.38	**0.39** (0.14)	**0.31** (0.04)	4	6	8	6721	746	183
*A. hahneli* (A)	0.62	**0.62** (0.14)	**0.32** (0.05)	4	6	8	14080	1523	361
*A. baeobatrachus* (A)	0.31	**0.42** (0.20)	**0.07** (0.04)	–	–	–	–	–	–
*D. tinctorius* (V)	0.46	**0.46** (0.14)	**0.53** (0.04)	2	3	4	8449	929	225
*R. margaritifera* (V)	1.00	**1** (0.003)	**0.19** (0.02)	8	11	14	2969	352	92
*A. flavescens* (V)	0.15	**0.16** (0.11)	**0.11** (0.05)	–	–	–	–	–	–
*R. lescurei* (V)	0.15	**0.17** (0.11)	**0.09** (0.05)	–	–	–	–	–	–

Naïve occupancy, i.e. the number of sites where the species was detected at least once during the survey is also indicated for each species.

### Number of Calling Males

We used N-mixture models to infer the number of calling males from repeated counts data. The number of calling males per transect was highest for *A. femoralis* (10.20+/−1.89 calling males per transect) followed by *A. hahneli* (5.83+/−2.60), *A. granti* (3.49+/−2.42) and *A. baeobatrachus* (1.49+/−0.94). For *A. femoralis*, we aimed to compare our estimation with a study conducted in the same area [26]. Most of the record for this species during the study (213 on 220) have been made in a strip of 40 m on each side of the transect. Using this value of 40 m, the number of calling males estimated for *A. femoralis* can be used to estimate the density of calling males as density = 10.2/(150*2*40) = 8.50+/−1.58 males/Ha.

The distance model with a half-normal key function had the best AIC score for both *A. femoralis* and *A. hahneli*. Using this key function, density estimates were 5.90+/−1.04 individuals/Ha for *A. femoralis* and 9.99+/−2.11 individuals/Ha for *A. hahneli*.

### Power Analysis

Due to the very low probabilities of detection estimated in this pilot study, *A. baeobatrachus* (probability of detection = 0.07+/−0.05), *A. flavescens* (probability of detection = 0.11+/−0.05) and *R. lescurei* (probability of detection = 0.09+/−0.05) were not taken into account in this power analysis. For the three remaining species with audio detection (*A. femoralis*, *A. granti* and *A. hahneli*), 8 survey occasions (K = 8, Table 4) were necessary to achieve a p’ = 0.95 (i.e. that the probability to detect the species at least once when it is present is equal to 0.95). For the two remaining species with visual detection (*D. tinctorius* and *R. margaritifera*), 4 survey occasions and 14 survey occasions (K = 4 for *D. tinctorius* and K = 14 for *R. margaritifera*, Table 4) were respectively necessary to achieve a p’ = 0.95.

**Figure 4:**
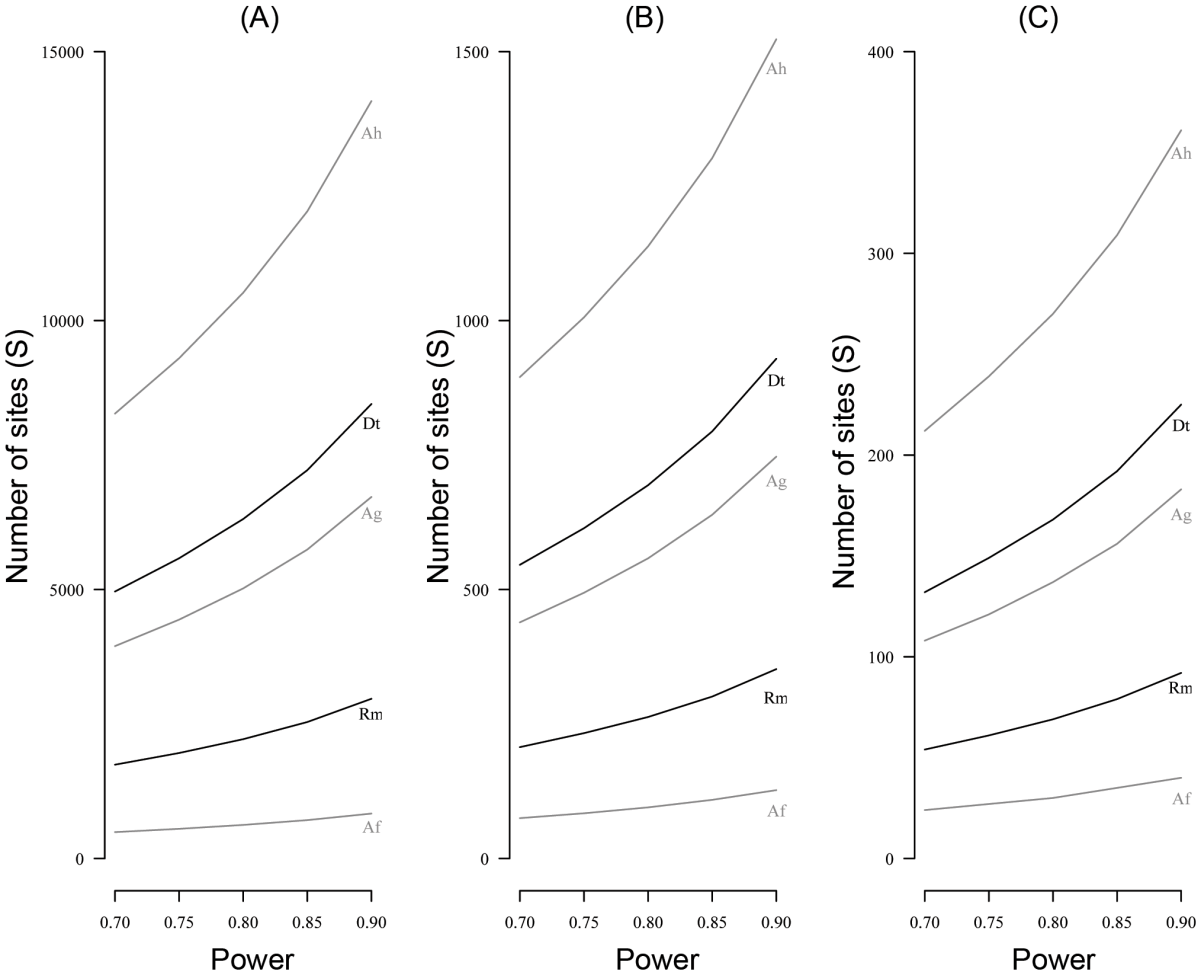
Number of sites to detect an occupancy decline with a given power. Number of sites (S) required to detect a decline for Af – *A. femoralis*, Rm – *R. margaritifera*, Ag – *A. granti*, Dt – *D. tinctorius*, Ah – *A. hahneli* with a given power for (A) R = 0.05, i.e. a decline of 5% between survey one and survey two, (B) R = 0.15 and (C) R = 0.30. Species with audio detection are indicated in grey and species with visual detection in black.

We therefore set the number of visit to K = 8 in order to determine the number of sites that need to be surveyed to detect a decline, considering that all the species will be surveyed simultaneously. This value of K implies that the probability to detect the species at least once when it is present (p’) will be equal to 0.95 for all species except *R. margaritifera* for which p’ will be equal to 0.8 (Table 4). The number of sites that would be required in a subsequent monitoring program to detect an occupancy decline with a given power varied considerably among species (Figure 4). Being the most widely distributed species in the study area, *A. femoralis* and *R. margaritifera* were the species requiring the fewest survey sites (40 and 92 sites respectively to detect a 30% decline with a power of 0.9). On the contrary, including *A. hahneli* would imply a very high number of sites (1523 sites to detect a 30% decline with a power of 0.9, see Figure 4). Irrespective of the species, detecting a 5% decline with presence/absence monitoring requires to survey a high number of sites (from 835 for *A. femoralis* to 14080 for *A. hahneli* to achieve a power = 0.9, Table 4).

## Discussion

Estimation of population size via CMR method is the most precise way to monitor population trend over time [31] but it is only suitable when the density of the population is high enough to ensure a sufficient recapture rate in a reasonable number of encounter events. In this study, only two species among eight were encountered frequently enough for using CMR models and only in habitats of high density despite a high number of encounter occasions. These two species are *D. tinctorius* (Dendrobatidae) and *R. margaritifera* (Bufonidae), the largest-bodied species of our eight focal species. We found that in patches where *D. tinctorius* occurs in high density, a stable estimation of population size may be achieved with 15 to 20 walks per transect, which is consistent with results from a previous study [32]. CMR estimates should therefore be preferred to other techniques for this species if the same sampling effort is possible. Moreover, in the future, we recommend concentrating capture events in a short period of time to minimize the risk of migration out of and into the population. For *R. margaritifera*, recapture rates remain low and yield a high coefficient of variation rendering CMR less suitable for this species.

The mean density of *D. tinctorius* estimated with our method was of 1097 individuals/ha. This high local density has been observed in other species of the genus *Dendrobates* like *D. pumilio*, which can reach 230 individuals/Ha in primary forest and 680 individuals/Ha in secondary forest on the Caribbean slope of Costa Rica [33]. This density is higher than found in a previous study in French Guiana (428 to 843 individuals/Ha, [32]). Nonetheless, the probability of occupancy of *D. tinctorius* was only 0.5 in the study site suggesting that this species can reach high density in specific habitats but is not distributed uniformly as it seems to be the case for *A. femoralis* and *R. margaritifera* (occupancy rates = 1 for these two species). Such considerations are important to properly assess the conservation status of this species. Indeed, this distribution pattern has also been reported at a more global scale in French Guiana, *D. tinctorius* being located only in elevated plateau. Moreover, this species can be infected by the fungus *Batrachochytrium dendrobatidis* in captive specimens [34] and in the wild, more specifically in this population [32], and efforts are needed to determine whether the fungus may have a significant impact on this species.

Using repeated count data of the number of simultaneously calling males per transects, we inferred the number of calling males in the transect. The *A. femoralis* population located in our study site is followed since 2007 [28] and the density estimated by [28] was 27 males/Ha and 18 females/Ha. Using a rough estimate of the area covered during the survey, we estimated that the density of calling males for *A. femoralis* was 8.50+/−1.58 males/Ha using repeated count data and 5.90+/−1.04 individuals using distance sampling. Our estimated density of calling males is smaller than estimation of male density obtained with an extensive search of all individuals in the patch. This discrepancy is likely due to the fact that in a given day, all the males are not simultaneously calling and we therefore cannot expect to detect all the reproductive males in the population. For example, a study in Peru showed that resident males of a Dendrobatidae species did not call daily and advertised their territories between 9.4 and 60% of days of territorial residence [35]. Moreover, the distance of detection may vary as a function of habitat type and meteorological conditions. Therefore the value of density estimated trough this method may be biased. Such method is therefore not suitable as a “stand-alone” technique but needs to be coupled with occupancy rate estimation. Moreover, the estimation of the number of calling males trough repeated counts data can be compared on a yearly basis and allow to detect a possible decline in the number of calling males. Future studies needs to be conducted to determine the relationship between the number of calling males in the population and the total population size and to test whether this relationship is stable over year.

In three of the four species for which audio detection was possible (*A. femoralis*, *A. hahneli* and *A. granti*), it led to a better detection probability than visual detection and should be preferred to monitor occupancy rates of species with day-calling males. For these three species, power analysis indicates that 8 visits are sufficient to ensure that the species will be detected at least once with a probability of 0.95 (p’ = 0.95). Estimation of occupancy rates with audio detection led to the same pattern of density than estimation using the maximum number of simultaneously calling males per transects. *A. femoralis* and *A. hahneli* are found to be the most common species while *A. granti* and *A. baeobatrachus* are less widely distributed in the study site. Such pattern of relative abundance and distribution needs to be linked in the future with the availability of suitable habitat for the species. Indeed, conservation status of a given species needs to be inferred from both the density of the species in suitable patches of habitat and from the availability and the risk faced by the suitable patches of habitat. For *A. baeobatrachus*, the detection probability was very low even with audio detection most likely due to the fact that very few individuals were still reproducing at the time of the study. We therefore recommend that audio sessions should be conducted preferentially at the beginning of the rainy season (from January to March).

For *Atelopus flavescens* and *Rhinella lescurei*, none of the three methods tested to follow population trends over time could be used with confidence, i.e. allowing to effectively monitor population trend over time. We did not use audio detection for these two species and visual surveys led to a very low detection probabilities and an estimation of the rate of occupancy that cannot be trusted even with a high number of survey occasions. Future studies should be conducted more specifically on these two species, making better use of what is known of their ecology. For instance *A. flavescens* males are calling during the day from isolated places along river streams and audio transects may be used for this species with transects located specifically along calling places. *R. lescurei* has been described recently [36] and little information is currently available concerning its biology and distribution. To increase the conservation status of this species it would be important to design a specific monitoring plan in known populations.

Given the occupancy rates and detection probabilities estimated in this pilot study, power analysis indicates that, irrespective of the species, a very high number of sites are required to detect a small decline (R = 5%) in the probability of occupancy (up to more than 14 000 sites for *A. hahneli*). When the decline is higher (R = 30%), the number of sites required to detect a decline in the probability of occupancy fall in a reasonable range (from 40 sites for *A. femoralis* to 392 sites for *A. hahneli*). Such presence/absence monitoring may not be useful to detect a small and rapid decline at a small spatial scale but should be implemented at a broader spatial scale. The design recommendations are based on the probability of detection and occupancy rates estimated in this pilot study and should therefore be re-evaluated as these change. Abundance-induced heterogeneity in detection probability can induce bias in the occupancy estimator [37]. If such heterogeneity is suspected, a model that accounts for it would be more appropriate.

In conclusion, the three methods of population size estimation tested in this study were found to be complementary and should be used in combination for different focal species (Table 1). With the prospect of a long-term study of amphibian species in French Guiana and more largely in the Guiana Shield, a specific protocol should be implemented. First, CMR is appropriate to monitor population sizes of *Dendrobates tinctorius* and *Rhinella margaritifera* in patches of high density. A notable advantage of this technique is that individuals caught for pattern identification could be also paired with a survey on the body condition of the individuals across years and on the presence of potential pathogens such as *Batrachochytrium dendrobatidis*. For dendrobatid species with day-calling males (*A. femoralis*, *A. granti*, *A. hahneli* and *A. baeobatrachus*), the use of audio transects distributed in the study zone and monitored once a year at the beginning of the rainy season (8 visits per session) was found to be the most suitable technique. The number of sites to be surveyed need to be adapted from the design procedure described in this pilot study in order to optimize surveys so that population declines can be identified more effectively. We believe that these findings will help create a vibrant network of collaborators in one of the most important areas of amphibian diversity worldwide.

## Supporting Information

Figure S1 **Distribution of the number of capture per distance classes for (A)** ***D. tinctorius*** **and (B)** ***R. margaritifera.***
(TIF)

Table S1 **Recapture rates for each species on each transect.**
(XLS)
