# Supplementary figures and images for: Monitoring Strategy for Eight Amphibian Species in French Guiana, South America
Source: PLoS One. 2013 Jun 28;8(6):e67486. doi: 10.1371/journal.pone.0067486 (PMC3696091; doi:10.1371/journal.pone.0067486)

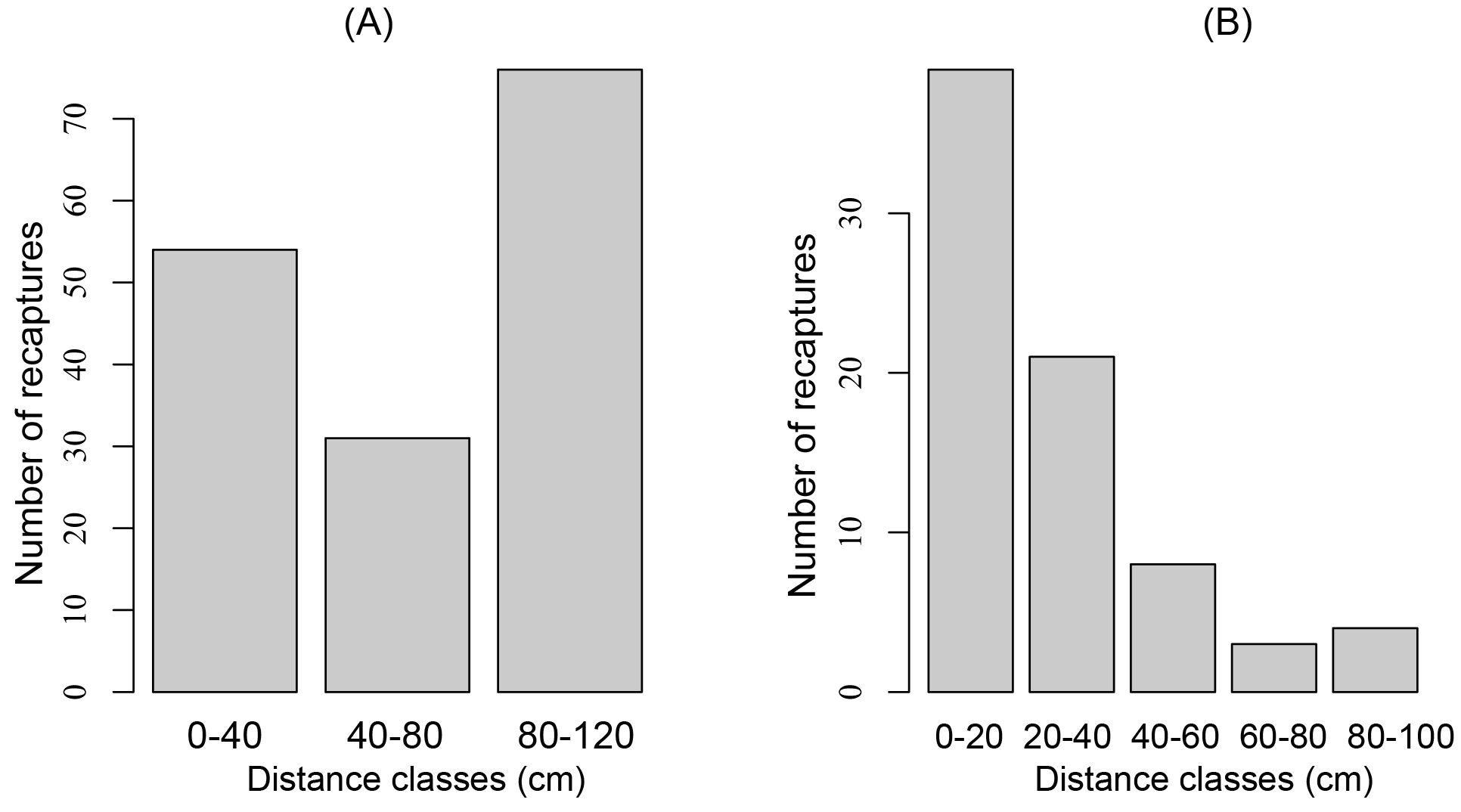

Supplement: Figure S1 — Distribution of the number of capture per distance classes for (A) D. tinctorius and (B) R. margaritifera. (TIF) [file pone.0067486.s001.tif]
